# Effect of extracellular vesicles derived from distinct brain cells on Aβ toxicity and assembly: focus on Microglia derived vesicles

**DOI:** 10.1186/2193-1801-4-S1-P17

**Published:** 2015-06-12

**Authors:** Pooja Joshi, Elena Turola, Ana Ruiz, Alessandra Bergami, Dacia Libera, Luisa Benussi, Clemens Falker, Paola Giussani, Giuseppe Magnani, Giancarlo Comi, San Raffaele, Giuseppe Legname, Roberta Ghidoni, Roberto Furlan, Michela Matteoli, Claudia Verderio

**Affiliations:** CNR Institute of Neuroscience, Italy; Department of Biotechnology and Translational Medicine, University of Milano, Italy; INSPE, Division of Neuroscience, San Raffaele Scientific Institute, Italy; Proteomics Unit IRCCS Istituto centro San Giovanni di Dio Fatebenefratelli, Italy; Institute of Neuropathology, University Medical Center, Germany; Scientific Institute, Italy,; Department of Neuroscience, SISSA, Italy; IRCCS Humanitas, Italy

**Keywords:** Extracellular Vesicles (EVs), Alzheimer’s Disease (AD), neurotoxicity

Alzheimer’s disease (AD) is a neurodegenerative disorder. The pathohistological features in AD are intracellular accumulation of neurofibrillary tangles and extracellular senile plaques. Plaque deposition leads to recruitment and activation of microglial cells, which induces neuroinflammation and drives neurodegeneration. Recent evidence show that soluble pre-fibrillar Aβ species, rather than insoluble fibrils, are highly neurotoxic and correlate with disease severity.Hence, preventing formation of soluble Aβ and its interaction with neurons is a major goal in AD. Despite massive efforts, how extracellular factors regulate assembly of Aβ peptide and neurotoxic activity of Aβ species is still largely undefined. Recent studies indicate that Extracellular Vesicles (EVs), including exosomes and PM-derived microvesicles (MVs), may influence Aβ neurotoxicity. Our findings reveal that production of microglial MVs (m-MVs) is strikingly high in patients with mild cognitive impairment and AD as compared to healthy controls and positively correlates with markers of neurodegeneration and hippocampal atrophy. Furthermore we found that MVs isolated from the CSF of AD patients are toxic to cultured hippocampal neurons. Through in vitro studies we demonstrate that the m-MVs promote generation of neurotoxic soluble species from almost inert Aβ aggregates, which is mediated by lipid components of MVs. Our findings suggest that m-MVs favor formation of neurotoxic Aβ species throughout the brain, possibly representing the mechanism behind transynaptic spread of Aβ in AD. On the other hand, studies conducted by Yuyama et al 2012, 2014 & An et al 2013 suggest that exosomes produced by neurons may exert opposite action by neutralizing neurotoxicity of soluble Aβ. To verify if the overall effect of exosomes and MVs on Aβ neurotoxicity may vary depending on parental cell type we are currently studying the influence of EVs (exosomes & MVs) derived from distinct brain cells on Aβ toxicity and assembly.

